# 

**Published:** 1919-01

**Authors:** 


### Thank You!

We hereby acknowledge receipt of subscriptions from the following persons:

Dr. J. A. Allison, Henrietta, Texas....Paid to June, 1919.

Dr. L. T. Baker, Dixie, La..........Paid to October, 1918.
Dr. J. F. Bell, Tyler, Texas..........Paid to April, 1920.
Dr. J. F. Bunkley, Seymour, Texas...Paid to November, 1919.
Dr. P. M. Butler, Tucson, Ariz. .....Paid to July, 1919.
Mrs. John T. Briscoe, Devine, Texas..Paid to December, 1918.
Dr. Hines Clark, Crowell, Texas.....Paid to January, 1919.
Dr. H. W. Cummings, Hearne, Texas.Paid to June, 1919.
Dr. W. H. Campbell, Nacogdoches...Paid to December, 1918.
Dr. Z. W. Casey, Albany, Texas.....Paid to July, 1919.
Dr. Augustus Davis, Bernalillo, N. M..Paid to June, 1919.
Dr. C. S. Diggs, Waxhachie, Texas....Paid to January, 1919.
Dr. W. M. Dodson, Woodsboro, Texas..Paid to June, 1919.
Dr. T. V. Horn, Philadelphia, Miss...Paid to July, 1919.
Dr. J. L. Haley, Lawn, Texas........Paid to December, 1918.
Dr. W. A. Harper, Austin, Texas....Paid to July, 1919.
Dr. J. M. Hons, San Marcos, Texas...Paid to July, 1919.
Dr. J. H. Hannabass, Snyder, Texas..Paid to July, 1919.
Dr. S. C. Grant, Mulberry, Ark. .....Paid to June, 1918.
Mr. R. W. Gardner, New York.......Paid to January, 1919.
Dr. Eusibio Guajardo, Monterey, N. L.Paid to June, 1919.
Dr. M. H. Logan, Bluffdale, Texas...Paid to October, 1919.
Dr. R. N. Miller, Beaumont, Texas..Paid to May, 1919.
Dr. J. H. Miller, Clyde, Texas........Paid to June, 1919.
Dr. G. L. Odom, Gretna, La. ........Paid to October, 1919.
Dr. J. R. S. Pitts, Waynesboro, Miss.Paid to February, 1919.
Surg.-General, U.S.A., Washington, D. C.Paid to June, 1919.
Dr. R. L. Spann, Dallas, Texas.......Paid to October, 1919.
Dr. A. Weaver, Cumberland, Iowa...Paid to December, 1919
Dr. F. A. Young, Montgomery, Texas.Paid to April, 1920.
Dr. A. Y. Easterwood, Ardmore, Ok..Paid to May, 1919.

### Deaths.

Died, December 31, 1918, Dr. R. H. Eanes of Round Rock, Texas.

Information has been received of the death in France of Dr.

H. Lee McNeal, of Houston, Texas. Dr. McNeal had the rank of captain in the medical corps and had been in France for more than a year, at the time of his death being in charge of an emergency hospital near the front.

Dr. George P. Smartt of Austin, died at the Austin City hospital on Thursday morning, December 26, after a brief illness of influenza.

During the influenza epidemic he waited upon more than three hundred cases of that disease, nearly two-thirds of the number being charity cases.

And after being taken sick, with fever of 105 degrees, Dr. Smartt got up out of bed and waited upon two cases when no other medical aid could be obtained for them. Literally he gave his life to his professional ideal of duty.

Dr. Robert A. Gray, aged 88, a native of Frankfort, Ky., died in Shreveport, La., November 25, after a brief illness. He was a veteran of the civil war, serving as surgeon with "Drew's battalion." After the war he returned to Shreveport. He was president of the first medical association in that State. He is survived by his wife and two sons.

Though his name has never appeared on a casualty list, and his death has not been officially confirmed, Dr. Knight W. Field, of Dallas, Texas, a first lieutenant in the Medical Corps, has been reported killed in action some time during October.

Dr. J. H. Perry of Canadian, died November 7, 1918, of pneumonia.

### Old Jokes That We Have Met.

A True Patriot—"Why don't you get an alienist to examine your son?"

"No, sir! An American doctor is good enough for me."—Baltimore American.

---

When He Stuttered—"Do you always stutter like that?" asked the doctor examining the recruit.

"N-no, sir," was the reply. "Only w-w-when I talk."

---

She—"Here's the paper says a lawyer told a man in the court that he was particeps criminis in the affair. What does that mean, William?"

He—"Well, my dear, you ought not to ask me to explain such things to you before the children."

---

Explicit—"Are you of the opinion, James," asked the slim-looking man of his companion, "that Dr. Smith's medicine does any good?"

"Not unless you follow the directions."

"What are the directions?"

"Keep the bottle tightly corked."

A Close Call.—Two young physicians in a Western city who were struggling to get a foothold in their profession met one day and exchanged views touching things of interest. Presently the talk turned to the last case one of them had handled.

"Yes," remarked the young medico, "the operation was just in the nick of time. In another twenty-four hours the patient would have recovered without it."—Harper's Magazine.

---

Quite Easy—"Well," asked the doctor, "how did you find yourself this morning?"

"Oh, easy enough," answered the patient. "I just opened my eyes and here I was."

---

Getting It—A lunger went to a druggist to get an empty bottle. Selecting one that answered his purpose, he asked:

"How much?"

"Well," said the clerk, "if you want the empty bottle it'll be one cent, but if you have something put in it we won't charge anything for the bottle."

"Sure, that's fair enough," observed Pat. "Put in a cork."

---

Could we judge all deeds by motives
That surround each other's lives,
See the naked heart and spirit,
Know what spur the action gives,
Often we would find it better
Just to judge all actions good;
We should love each other better
If we only understood.

---

No Doctor Needed.—"Did the doctor put you on a diet?"

"He didn't have to—Hoover did it."

---

New Kind of Nourishment.—An old darky was sent to a hospital. One of the gentle sisters put a thermometer in his mouth to take his temperature. Presently, when the doctor made his rounds, he said:

"Well, Nathan, how do you feel?"

"I feel right tol'ble, boss."

"Have you had any nourishment?"

"Yassir."

"What did you have?

"A lady done give me a piece of glass ter suck, boss."

CASE OF MUMPS DRIVES CROWD FROM ELEVATOR.—An elevator in a tall office building which apparently was packed to capacity stopped at the twelfth floor and took on another passenger. A friend of the newcomer exclaimed: "What's the matter, Bill? Have you got the mumps or are you getting fat?"

"Jim, I've got the mumps," was the answer. "Just been up to see a doctor, who told me to go home and go to bed."

"Let me off at the next floor," a woman said.

"At the next floor," which happened to be the fifth, every person on the elevator got off, except the man with the mumps and the operator. And everyone of them seemed to be in a hurry.

---

A FAILURE—"Did you get acclimated when you went to Cuba?"

"Yes, and by the best doctor I could find, but it didn't take."

